# Impact of Treadmill Running and Sex on Hippocampal Neurogenesis in the Mouse Model of Amyotrophic Lateral Sclerosis

**DOI:** 10.1371/journal.pone.0036048

**Published:** 2012-04-25

**Authors:** Xiaoxing Ma, Mazen J. Hamadeh, Brain R. Christie, Jane A. Foster, Mark A. Tarnopolsky

**Affiliations:** 1 Medical Sciences, McMaster University, Hamilton, Canada; 2 Department of Medicine, McMaster University, Hamilton, Canada; 3 Department of Pediatrics, McMaster University, Hamilton, Canada; 4 Kinesiology and Health Science, York University, Toronto, Canada; 5 Medical Sciences, University of Victoria, Victoria, Canada; 6 Department of Psychiatry and Behavioural Neurosciences, McMaster University, Brain-Body Institute, St. Joseph's Healthcare, Hamilton, Ontario, Canada; University of South Florida, United States of America

## Abstract

Hippocampal neurogenesis in the subgranular zone (SGZ) of dentate gyrus (DG) occurs throughout life and is regulated by pathological and physiological processes. The role of oxidative stress in hippocampal neurogenesis and its response to exercise or neurodegenerative diseases remains controversial. The present study was designed to investigate the impact of oxidative stress, treadmill exercise and sex on hippocampal neurogenesis in a murine model of heightened oxidative stress (G93A mice). G93A and wild type (WT) mice were randomized to a treadmill running (EX) or a sedentary (SED) group for 1 or 4 wk. Immunohistochemistry was used to detect bromodeoxyuridine (BrdU) labeled proliferating cells, surviving cells, and their phenotype, as well as for determination of oxidative stress (3-NT; 8-OHdG). BDNF and IGF1 mRNA expression was assessed by *in situ* hybridization. Results showed that: (1) G93A-SED mice had greater hippocampal neurogenesis, BDNF mRNA, and 3-NT, as compared to WT-SED mice. (2) Treadmill running promoted hippocampal neurogenesis and BDNF mRNA content and lowered DNA oxidative damage (8-OHdG) in WT mice. (3) Male G93A mice showed significantly higher cell proliferation but a lower level of survival vs. female G93A mice. We conclude that G93A mice show higher hippocampal neurogenesis, in association with higher BDNF expression, yet running did not further enhance these phenomena in G93A mice, probably due to a ‘ceiling effect’ of an already heightened basal levels of hippocampal neurogenesis and BDNF expression.

## Introduction

Throughout life, new neurons are generated in the sub-ventricular zone (SVZ) of the lateral ventricle and in the sub-granular zone (SGZ) of the dentate gyrus (DG) in the hippocampus [1]–[6]. Hippocampal neurogenesis is highly regulated by physiological factors, such as physical activity, and pathological processes, such as brain injury and neurodegenerative diseases [5]. Both voluntary wheel running and forced treadmill running have repeatedly been reported to promote adult hippocampal neurogenesis [7]–[10] and improve learning and memory [7], [11]. In contrast, impaired hippocampal neurogenesis has been linked with normal aging, radiation, and chronic alcohol exposure; which are associated with oxidative stress, and the imapirment can be rescued by physical exercise [12]–[16]. However, the role of oxidative stress as a mediator of hippocampal neurogenesis and/or its response to exercise or neurodegenerative diseases remains controversial. For example, in animal models of Alzheimer's disease (AD), both enhanced or impaired hippocampal neurogenesis have been reported [17], [18]. Furthermore, voluntary running failed to rescue impaired hippocampal neurogenesis in the R6/2 mouse model of Huntington's disease (HD) [19].

Sex differences in adult neurogenesis may contribute to variability reported in some studies [20], [21]. This difference is dependent on the estrogen status of the female, as only proestrus female rats (with high estradiol levels) show higher levels of cell proliferation than males [20]. However, female meadow voles exhibit higher levels of cell proliferation than males only during the non-breeding season (when estradiol levels are low) [21]. Furthermore, reproductively active female meadow voles with high endogenous levels of estradiol have suppressed rates of cell proliferation in the DG compared with reproductively inactive females with low estradiol, yet more new cells survived in females with high endogenous levels of estradiol [22].

Amyotrophic lateral sclerosis (ALS), also known as Lou Gehrig's disease, is a motor neuron degenerative disease strongly associated with heightened oxidative stress [23], characterized by selective loss of motor neurons in the spinal cord, brain stem, and cerebral cortex. Oxidative injury has been shown in the parietal cortex and cerebellum, regions that are typically clinically unaffected in the early stages of ALS, suggesting widespread oxidative stress [24]. The G93A mouse has a transgenic over-expression of a mutation in Cu/Zn-superoxide dismutase (SOD1), associated with hereditary ALS (glycine substitution to alanine at amino acid 93, G93A). Overexpression of mutant SOD1 in G93A mice causes a progressive paralytic disease, which resembles human ALS in clinical and pathological features [25]. In G93A mice, elevated oxidative stress in the brain has been reported [26]–[28]. In addition, sex has been proposed as one of the possible modifying factors in ALS [29] and G93A mice. In G93A mice, our and other laboratories found that there is a sex difference in the onset and progression of diseases, and, female and male mice respond differently to exercise training [30], [31]. In the current study, we employed G93A mice to investigate the influence of oxidative stress, exercise, and sex on hippocampal neurogenesis.

The molecular mechanisms underlying adult neurogenesis are not completely understood; however, growth factors are clearly implicated. Brain-derived neurotrophic factor (BDNF) plays a role in the maintenance of basal levels of hippocampal neurogenesis [32]–[34]. The up-regulation of hippocampal BDNF has been reported in neurogenesis following exercise [35], [36]. Importantly, BDNF could interact with other factors, such as serotonin and reactive oxygen species (ROS), to promote proliferation, differentiation and survival of new neurons. For example, nitric oxide (NO) has been reported to act in a positive feedback loop with BDNF to promote proliferation and differentiation [37]. In addition, insulin-like growth factor 1 (IGF1) is a growth promoting peptide hormone produced both centrally in neurons as well as glial cells [38]. By binding to its receptor, type-1 IGF receptor (IGF-1R), IGF1 activates several growth and survival-promoting intracellular signaling pathways, including the MAPK and PI3K/Akt pathways [39], [40]. As well, IGF1 promotes hippocampal neurogenesis and is involved in physical activity induced hippocampal neurogenesis [41], [42].

To date, there is a paucity of information regarding hippocampal neurogenesis in G93A mice. While one study reported lower cell proliferation in DG with no change in neurogenesis in the hippocampus and spinal cord in 16-week-old symptomatic G93A mice [43]; two other studies showed increased neurogenesis in the spinal cord in this model [44], [45]. We, and others, have shown that sex and exercise have independent and interactive effects on disease progression and onset in the G93A mice [30], [31]. The aims of this study were to: (1) examine the basal level of hippocampal neurogenesis and the impact of exercise and sex on hippocampal neurogenesis in G93A mice, an animal model of heightened oxidative stress; (2) investigate whether BDNF and IGF1 are involved in the regulation of basal levels of hippocampal neurogenesis and the response to exercise in G93A mice; and (3) determine whether oxidative stress *per se* is a regulator for the hippocampal neurogenesis in G93A mice.

## Materials and Methods

### Animals and Experimental Design

Male transgenic G93A mice (B6SJL-TgN[SOD1-G93A]1Gur) were purchased from the Jackson Laboratory (Bar Harbour, ME) and harem bred with female wild-type B6SJL mice, and a colony was established. All mice used in the current study were from the F1 generation of this breeding to avoid the potential for random variance in transgene copy number. Offspring were genotyped for the G93A transgene using PCR of DNA extracted from tail samples as outlined by Jackson Laboratories. Animals were housed five per cage before 50 days of age and 1 per cage after 50 days of age with a 12-h light/dark cycle. All mice were fed standard murine chow and water *ad libitum*, and food intake was recorded weekly for each cage. The experimental protocol was approved by the McMaster University Animal Research Ethics Board and was carried out in accordance with guidelines of the National Institutes of Health and the Canadian Council on Animal Care. At 40 days of age, G93A and wild-type (B6SJL) mice were randomly divided into the cell proliferation study group (N = 46, 5–6/group) and the cell survival study group (N = 92, 9–13/group) stratified according to exercise training status and sex (see below). Starting at 50 day of age, mice were housed to 1 per cage, and body weight, body condition, ability to move, and clinical score were recorded once a week until mice were sacrificed. In the cell proliferation group, mice at 90 days of age were injected for seven consecutive days with bromo-deoxyuridine (BrdU) and were subjected to treadmill running for one week (see below) or to a sedentary lifestyle. Twenty-four hours after the last BrdU administration, mice were sacrificed and brains were collected to quantity BrdU-labeled cells in the hippocampus by immunohistochemistry (IHC) for cell proliferation. In the cell survival group, mice at 80 days of age were injected for seven consecutive days with BrdU and were subjected to treadmill exercise for four weeks or to a sedentary lifestyle. Three weeks after the last administration of BrdU, mice were sacrificed to examine BrdU-labeled surviving cells by IHC, cell differentiation (cell fate determination of BrdU labeled surviving cells) by immunofluorescence staining, mRNA expression of BDNF, IGF1, SOD2, and catalase by *in situ* hybridization, and, markers of oxidative stress (3-NT; 8-OHdG) by IHC.

### BrdU injection

BrdU (Sigma, St. Louis, MO) was dissolved in fresh 0.9% NaCl and sterile-filtered through a 0.2 µm filter. Each mouse received one single dose (50 mg/kg) at a concentration of 1 mg/ml, one intraperitoneal injection per day for seven consecutive days.

### Exercise training

#### Cell proliferation exercise training

Exercise training consisted of four sessions over a one week period. In the first and second training session, the mice were acclimatized to the treadmill, running at 15 m/min for 30 min. In the third and fourth training session, the exercise duration was 45 min at 15 m/min.

#### Cell survival and cell differentiation exercise training

Exercise training lasted for four weeks, 3 times a week. In the first and second weeks, the mice were acclimatized to the treadmill, running at 15 m/min for 30 min. In the third and forth weeks, the duration of training reached 45 min at 15 m/min.

### Tissue preparation

Mice were anesthetized with isoflurane inhalation and perfused transcardially with 50 mL of 0.02 M phosphate buffered saline (PBS), followed by 50 ml of 4% paraformaldehyde (PFA). Brains were removed and fixed with 4% PFA at 4°C overnight, transferred into a 30% sucrose solution until saturated (24 hours), and embedded in OCT and stored at −80°C until sectioning. The cryostat was used to cut sections. In the cell proliferation group, brains were cut to coronal sections (40 µm /section) throughout the entire rostral-caudal extent of the hippocampus (Bregma −0.94∼−3.88 mm) for BrdU IHC [46]. In the cell survival study group, half hemisphere of brains was cut into coronal sections (40 µm/section) throughout the entire rostral-caudal extent of the hippocampus (Bregma −0.94∼−3.88 mm) for BrdU IHC and immunofluorescence staining [46]. The other half of brains was cut to sagittal sections (16 µm/section) throughout the extent of the hippocampus (Lateral 0.72∼2.28 mm), collected in gelatin-coated slides, and kept at −80°C for *in situ* hybridization and IHC [46]. Coronal sections were stored at −20°C in cryoprotectant containing 25% glycerin, 25% ethylene glycol, and 0.05 M phosphate buffer.

### IHC for detection of BrdU-labeled cells

A one in six series of sections throughout the entire rostral-caudal extent of the hippocampus was used to assess the number of BrdU-labeled cells. Staining was carried out on free-floating sections as previously described [8]. Briefly, free-floating sections were washed with Tris-buffered saline (TBS) and treated with 0.6% H_2_O_2_ in TBS for 30 min to block endogenous peroxide activity. Sections were then incubated for 2 h in deionized 50% formamide/50% 2× SSC buffer (0.3 M NaCl/0.03 M sodium citrate) at 65°C, rinsed in 2×SSC (5 min), incubated in 2 N HCl at 37°C (30 min), and then placed in 0.1 M boric acid (pH 8.5, 10 min). Sections were then rinsed in TBS (6×10 min), incubated in TBS++ (TBS, 0.1% Triton X-100 and 3% normal donkey serum) for 30 min and then incubated in mouse anti-BrdU monoclonal antibody (1∶200, Chemicon, Temecula, USA) for 12 h at 4°C. After being rinsed with TBS, sections were immersed in biotinylated donkey anti-mouse antibody (1∶500, Chemicon, Temecula, USA) for 2 h at 4°C. Vectastain Elite ABC kit (Vector Laboratories, Burlingame, USA) and diaminobenzidine (DAB) kit (Vector Laboratories) were used to visualize BrdU-positive cells. Finally, sections were mounted on gelatin-coated slides, air dried overnight, counterstained with 0.1% cresyl violet staining, dehydrated in graded ethanol and xylene, and coverslipped using permount. The number of BrdU-labeled cells in the DG was examined using light microscopy.

#### Cell counting of BrdU-labeled cells

BrdU-labeled cells were counted in every section of a one-in-six series (240 µm apart) throughout the rostral-caudal extent of the subgranule cell layer (defined as the area ∼20 µm between granule cell layer and the hilus). Because we were interested in relative differences and not necessarily an absolute value of BrdU-labeled cells in DG, BrdU positive cells in the entire DG were quantified by profile-sampling methods [47]. In the x-y plane, BrdU positive cells limited to the subgranule cell layer were manually counted in a blind fashion at 40× magnification (Olympus, BX60, Center Valley, USA). For the z-plane, a modified optical dissector method was employed that excluded immuno-labeled cells on the uppermost surface of the slice. BrdU positive cells in the DG in both (cell proliferation group) or one hemisphere (cell survival group) was counted for each section.

The corresponding sample area of the DG granule cell layer was outlined and determined by using image analysis software (Image-Pro software). Data were expressed as cell density (cells/mm^2^) per DG of BrdU-labeled cells.

### Immunofluorescence for cell differentiation

Immunofluorescent triple labeling for BrdU, neuronal nuclei (NeuN) and glial fibrillary acidic protein (GFAP) was done on free floating sections as previously described [8]. Briefly, after pretreatment in deionized formamide for 2 h at 65°C, 2 N HCl for 30 min at 37°C, and 3% normal goat serum (Vector Laboratories) for 30 min at room temperature, sections were incubated in a cocktail of rat anti-BrdU (1∶100, Serotec, Martinsried, Germany), mouse anti-NeuN monoclonal antibody (1∶500, Chemicon, Temecula, USA), and chicken anti-GFAP polyclonal antibody (1∶200, Chemicon, Temecula, USA) overnight at 4°C. Next day, sections were rinsed in TBS, blocked in 3% normal goat serum and then incubated in a cocktail of Alexa Fluor 488 goat anti-rat antibodies (1∶500, Molecular Probes, Carlsbad, USA), Alexa Fluor 568 goat anti-mouse highly cross-adsorbed antibody (1∶500, Molecular Probes, Carlsbad, USA), and Alexa Fluor 647 goat anti-chicken antibody (1∶500, Molecular Probes, Carlsbad, USA) for 4 h at 4°C. Then sections were rinsed several times, mounted on gelatin-coated slides, coverslipped with SlowFade Gold antifade reagent (Molecular Probes, Carlsbad, USA) and examined by confocal microscopy.

#### Analysis of cell phenotype

A one-in-six series of sections from mice surviving 3 wk after the last injection of BrdU was triple labeled as described above and analyzed by confocal microscopy (Leica TCS SP5, Germany). Fifty of BrdU positive cells per animal (n = 5∼6 per group) were analyzed for co-expression of BrdU and NeuN for neuronal phenotype and GFAP for glial phenotype. Co-labeling was verified throughout the z-axis of focus. The percentage of BrdU+ cells co-labeled with NeuN, with GFAP, or without NeuN or GFAP was determined.

### In Situ Hybridization for mRNA expression

#### Construction of cRNA probes

Total RNA was isolated from pooled muscle of C57B6 mice. RT-PCR was performed to amplify cDNA fragments. For mouse IGF1, forward 5′–TGG ATG CTC TTC AGT TCG TG-3′ and reverse 5′-TCC TGC ACT TCC TCT ACT TGT-3′ primers were used to amplify a 318 bp cDNA fragment. For mouse SOD2, forward 5′-CGC CAC CGA GGA AAG TA-3′ and reverse 5′-CAG TCA TAG TGC TGC AAT GC-3′ primers generated a 559 bp cDNA fragment. For mouse catalase, forward 5′-GCT ATG GAT CAC ACA CCT T-3′ and reverse 5′ -GTT CAC AGG TAT CTG CAG-3′ primers generated a 488 bp cDNA fragment. After being purified, PCR products were cloned into pGEM-T easy vector (Promega Biotech, Madison, WI) and sequenced to verify their identities. The vector containing rat full-length BDNF cDNA insert (gift of Drs. J. Lauterborn and C. Gall, University of California Irvine, Irvine, CA) or 318 bp cDNA fragment of mouse IGF1 or 559 bp cDNA fragment of mouse SOD2 or 488 bp cDNA fragment of mouse catalase were used as templates for riboprobe synthesis. The antisense and sense riboprobes were synthesized using the Riboprobe System (Promega Biotech, Madison, WI) with α-35S-UTP (specific activity >1,000 Ci/mmol; Perkin Elmer, Boston, MA) and T3 or T7 RNA polymerase (Promega Biotech, Madison, Wisconsin). The transcribed products were purified by using ProbeQuant G-50 Micro Columns (GE Healthcare Bio-Sciences Corp, Piscataway, NJ) and probe labeling was determined by scintillation counting.

In situ hybridization was performed as previously described [48]. Briefly, brain sections were fixed with 4% formaldehyde, acetylated with acetic anhydride, dehydrated in graded ethanol, delipidated with chloroform, and air dried. Radioactive-labeled probes were diluted in a hybridization buffer and applied to brain sections (∼500,000 CPM/section). Hybridization was carried out overnight at 55°C in a humidified chamber. After hybridization, RNase treatment, high-stringency post hybridization washes, and dehydration were performed. Slides and ^14^C plastic standards were placed in X-ray cassettes, apposed to film (BioMax MR; Eastman Kodak, Rochester, NY) for 10 days, and developed in an automatic processor.

Quantitative analysis of autoradiograms was done using a Macintosh computer-based image-analysis system with NIH Image software (http://rsb.info.nih.gov/nih-image). Autoradiographic film images were captured during one session with constant settings of camera (Q Capture v1.2.0, Q Imaging Corporation, Surrey, Canada) and light (Northern Light, Model-R95, Imaging Research Inc, St. Catharines, Canada). NIH Image software was used to construct the calibration curve of the [^14^C] standards and to quantify the signal at the DG. Then, contours were drawn over the DG and optical densities were automatically measured from the corresponding regions of the autoradiographic images.

### IHC for detection of SOD2, catalase, 8 hydroxy-2-deoxyguanosine (8-OHdG), and nitrotyrosine (3-NT)

Sagittal brain sections were fixed with 4% formalin for 10 min and blocked with normal goat serum, avidin, biotin, and, 0.03℅ hydrogen peroxide. The primary antibodies, including rabbit anti-mouse SOD2 (1∶600 dilution, Abcam, Cambridge, UK), rabbit anti-mouse catalase (1∶1000 dilution, Abcam, Cambridge, UK), rabbit anti-mouse 8-OHdG (1∶400, Secrotec, Martinsried, Germany), and rabbit anti-mouse 3-NT (1∶200, Upstate, Billerica, USA) were then applied and incubated overnight at 4°C. The following day, the secondary antibodies, including biotinylated goat anti-rabbit IgG (1∶500, Jackson lab, Bar Harbor, USA) were applied and incubated for one hour at room temperature followed by one hour incubation with streptravidin-horseradish peroxidase. Slides were developed for 5–10 min in DAB, dehydrated, and mounted with permount.

Quantitative analysis of optical density was done using a Macintosh computer-based image-analysis system with NIH Image software. Black-and-white images were captured during one session with constant settings of camera. NIH Image software was used to draw contours over the DG and optical densities were automatically measured from the corresponding regions of images.

### Statistical analysis

Data were analyzed based on our *planned comparisons* to answer the following questions: a) Are there any differences in the outcome measures at the basal sedentary levels between the G93A and WT mice? b) Are there any effects of activity and sex within each genotype variant? To address these main questions, we used a two-way analysis of variance (ANOVA) (Statistica, version 6.0, StatSoft, Tulsa, OK) to determine significant differences a) in the sedentary mice, with the two factors being genotype (G93A vs. WT) and sex (male vs. female), b) in the WT mice, with the two factors being activity (EX vs. SED) and sex (male vs. female), and c) in the G93A mice, with the two factors being activity (EX vs. SED) and sex (male vs. female). When there was significant difference, Tukey's honestly significant difference test was used post-hoc to determine the source of difference. Based on the hippocampal changes in G93A mice described above, including higher oxidative stress [26], [49], higher growth factor content [50], [51], activation of ERK pathway [52], higher hippocampal dependent function [53], and increased cell proliferation and neurogenesis in the spinal cord of G93A mice [44], [45], we *a priori* hypothesized that G93A mice would have a higher basal level of hippocampal neurogenesis compared to WT mice. In addition, due to extensive evidence showing that exercise promotes hippocampal neurogenesis under normal wild-type conditions [8], [54], [55] and possibly in neurodegenerative disease, we *a priori* hypothesized that exercise would promote neurogenesis both in WT and G93A mice. Furthermore, due to the evidence that estrogen up-regulates hippocampal neurogenesis [56] and that there is a sex difference in clinical aspects of ALS demographics and G93A mice [31], we *a priori* hypothesized that female mice would show greater hippocampal neurogenesis versus male mice. And based on the evidence that BDNF and IGF1 play a role in basal hippocampal neurogenesis [32] and up-regulation of hippocampal neurogenesis following exercise [57]–[59], we *a priori* hypothesized that BDNF and IGF1 would be involved in basal level of hippocampal neurogenesis in G93A mice with exercise increasing hippocampal neurogenesis in association with higher levels of BDNF and IGF1 in WT and G93A mice. Finally, based on evidence that higher oxidative stress was observed in motor neuron areas [60]–[62] and non-motor neuron areas in G93A mice [26], [49], we *a priori* hypothesized that markers of oxidative stress and antioxidant enzymes would increase to compensate for elevated oxidative stress in the hippocampus. Hence, for these specific analyses, a 1-tailed test was used. For all other analyses, a 2-tailed test was used. Unless otherwise noted, all data are presented as means ± standard error of the mean (SEM). Significant differences were defined as *P*≤0.05.

## Results

### Hippocampal Neurogenesis

#### Cell Proliferation

**Figure 1:**
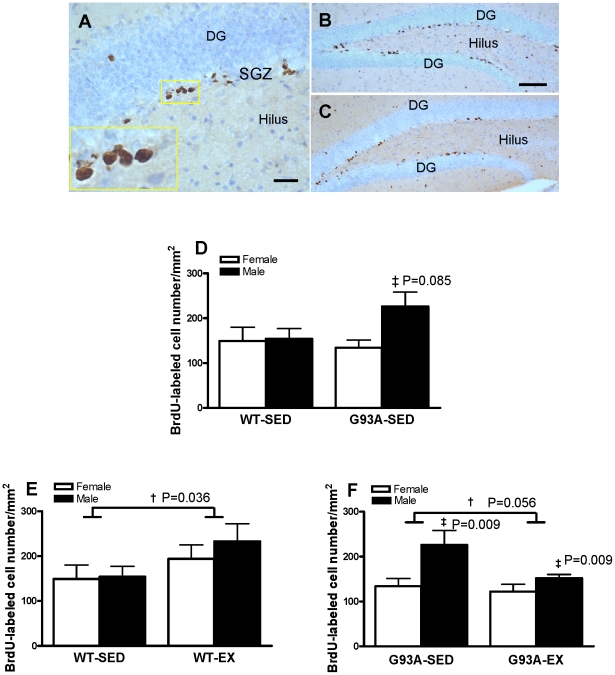
BrdU-labeled proliferating cells in the dentate gyrus (DG) of wildtype (WT) and G93A mice subject to treadmill running (EX) or sedentary lifestyle (SED). (A) A representative image showed that the majority of the BrdU-labeled proliferating cells in WT mice were located in the subgranular zone (SGZ), typically appearing in clusters and having an irregular shape with dense and homogenous staining of the nuclei (insert). Representative images showed BrdU labelled proliferating cells in WT sedentary mice (B) and in G93A sedentary mice (C). (D) G93A mice had 18.5% more proliferating cells than WT mice collapsed across sex, due to 68.7% greater number of proliferating cells in G93A males vs G93A females (‡ a trend, G93A-Male-SED>G93A-Female-SED, P = 0.085, n = 6 per group). (E) WT-EX mice had 42.4% more proliferating cells than WT-SED mice collapsed across sex. † WT-EX>WT-SED, P = 0.036, n = 5–6 per group. (F) G93A-EX mice had a trend to have 24.4% fewer proliferating cells vs SED mice. † G93A-EX<G93A-SED, a trend, P = 0.056. Meanwhile, G93A male mice had 50.0% more proliferating cells than G93A female mice. **‡** G93A male>G93A female, P = 0.009, n = 6 per group except for G93A EX males = 5. Data are means ± SEM. Scale bar = 25 µm in A, 100 µm in B,C.

Cell proliferation was determined as the number of BrdU labeled cells in the DG 24 h after the last BrdU injection. The majority of the BrdU-labeled cells were located in the SGZ and less frequently in the hilus (Figure 1A), typically appearing in clusters and showing an irregular shape with dense and homogenous staining of the nuclei (Figure 1A insert). The appearance and general distribution of BrdU-labeled cells did not differ between WT mice (Figure 1B) and G93A mice (Figure 1C). To examine the baseline level of cell proliferation in G93A mice, we compared the number of BrdU labeled cells between G93A-SED and WT-SED mice. While no significant difference was detected between genotypes, G93A male SED mice showed a trend to have 68.7% more BrdU-labeled cells than G93A female SED mice (226±32/mm^2^ vs 134±17/mm^2^; P = 0.085) (Figure 1D). For the WT mice, exercise training led to 42.4% more proliferating cells in the DG vs. SED (215±25/mm^2^ vs 151±19/mm^2^, P = 0.036) (Figure 1E). Whereas, for the G93A mice, exercise training strongly tended towards 24.4% fewer proliferating cells in the DG vs. SED (136±10/mm^2^ vs 180±22/mm^2^; P = 0.056) (Figure 1F). G93A male mice had more proliferating cells than G93A female mice in both SED and EX conditions (Figure 1F). Overall, in G93A mice, a) baseline level of cell proliferation was not different vs. WT mice, b) treadmill exercise showed a trend toward reduced cell proliferation, and c) a sex difference in the cell proliferation was present, with G93A males having significantly higher cell proliferation as compared with females.

#### Cell Survival

**Figure 2:**
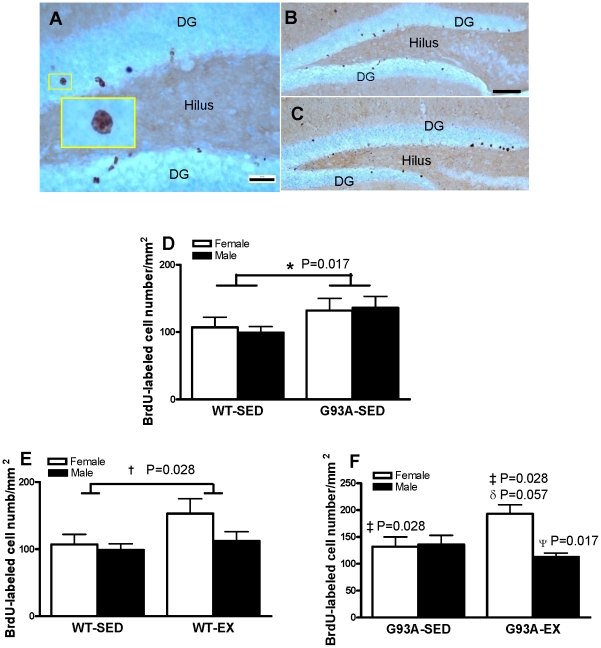
BrdU labelled surviving cells in the dentate gyrus (DG) of wildtype (WT) and G93A mice subject to treadmill running (EX) or sedentary lifestyle (SED). (A) A representative image showed that most surviving cells in WT mice were located in the DG, with more rounded nuclei, sometimes with the typical chromation structure of granular cells (insert). Representative images showed surviving cells in WT sedentary mice (B) and G93A sedentary mice (C). (D).G93A mice had 30.1% more surviving cells than WT mice collapsed across sex. *G93A-SED>WT-SED, P = 0.017, n = 10–12 per group. (E). WT-EX mice collapsed across sex had 29.1% more surviving cells than WT-SED mice. † WT-EX>WT-SED, P = 0.028., n = 10–12 per group. (F). Male G93A mice had 22.4% fewer surviving cells than female G93A mice (‡ G93A-M<G93A-F, p = 0.028), due mainly to the G93A-EX males having 41.5% fewer surviving cells than G93A-EX females (^Ψ^ G93A-Male-EX<G93A-Female-EX, P = 0.017). G93A female EX mice had a trend to have 46% more surviving cells than G93A female SED mice (δ a trend, G93A-Female-EX>G93A-Female-SED, P = 0.057), n = 10 per group except for G93A-SED females = 11. Data are means ± SEM. Scale bar = 25 µm in A, 100 µm in B,C.

Three weeks after the last injection of BrdU, cell survival of BrdU-labeled newborn cells was assessed in all mice [63]–[65]. Most BrdU-positive cells were located in the DG (Figure 2A). These cells had rounded nuclei, sometimes with the typical chromation structure of granule cells (Figure 2A insert). Figure 2B and 2C show representative images of surviving cells in WT and G93A mice, respectively. Sedentary G93A mice had 30.1% more surviving BrdU-positive cells compared to sedentary WT mice (134±12/mm^2^ vs 103±8/mm^2^; P = 0.017) (Figure 2D). For the WT mice, there were significantly 29.1% more BrdU-positive cells following exercise training vs. SED (133±14/mm^2^ vs 103±8/mm^2^, p = 0.028) (Figure 2E). For the G93A mice, females tended to have 46% more BrdU-positive cells following exercise training vs. SED (193±27/mm^2^ vs. 132±18/mm^2^, P = 0.057). Overall, male G93A mice had 22.4% fewer surviving cells than female G93A mice (125±10/mm^2^ vs 161±17/mm^2^, P = 0.028); however, this was strongly influenced by the fact that the male G93A mice had 41.5% fewer surviving cells than G93A females following exercise.

#### Cell Differentiation

**Figure 3:**
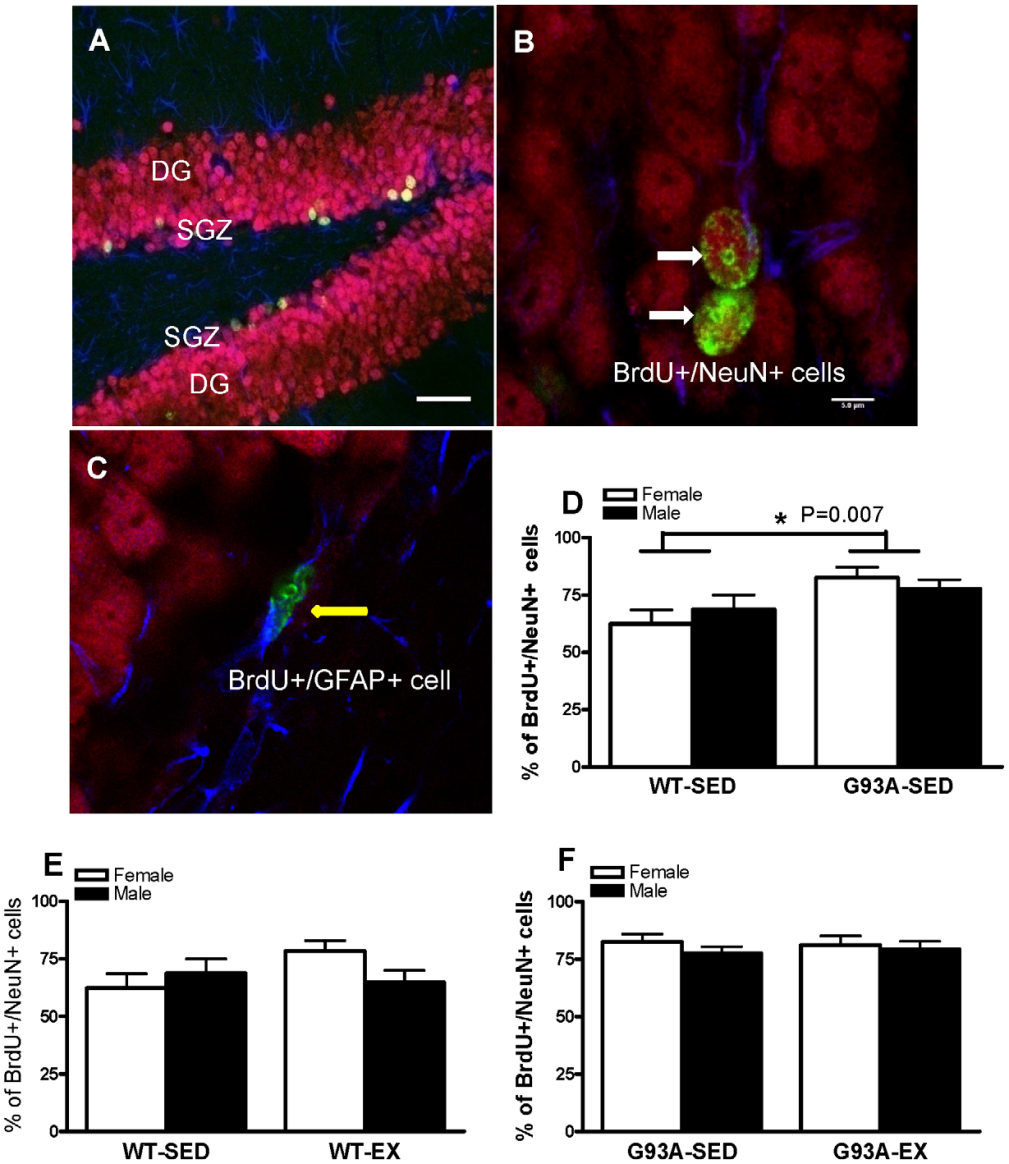
The cell phenotype of BrdU labeled surviving cells in the DG of WT and G93A mice subject to treadmill running (EX) or sedentary lifestyle (SED). (A) Representative confocal microscopic images of triple fluorescent staining in the DG of the hippocampus in WT mice. Green: BrdU, red: NeuN, Blue: GFAP. (B) A representative confocal microscopic image showed two newly generated neurons (white arrows) colocalized with BrdU and NeuN in WT mice. (C) A representative confocal microscopic image showed one newly generated astrocyte (yellow arrow) colocalized with BrdU and GFAP in WT mice. (D) The percentage of double labelled BrdU and NeuN cells was 22.4% higher in G93A-SED than in WT-SED collapsed across sex. *G93A-SED>WT-SED, P = 0.007, n = 5–6 per group. (E)There was no significant difference in the percentage of BrdU and NeuN double labelled cells between WT-EX and WT-SED mice, n = 5–6 per group. (F) There was no significant difference in the percentage of BrdU and NeuN double labelled cells between G93A-EX and G93A-SED mice, n = 5–6 per group. Data are means ± SEM. Scale bar = 100 µm in A; 5 µm in B and C.

Co-localization of BrdU positive staining (green color) with neuronal marker NeuN (red color) and astrocytic marker GFAP (blue color) was employed to determine the phenotype of newborn cells in the DG 3 wk after the last injection of BrdU. A representative confocal microscopic image of triple staining in Figure 3A shows red granule cells (neurons) stained with NeuN in the DG and blue cells (astrocytes) stained with GFAP in the hilus and molecular layer. Several orange cells (merged green and red colors) double stained with BrdU and NeuN in SGZ (Figure 3A). Newly generated neuronal cells were double stained with green (BrdU positive) and red (NeuN positive) (Figure 3B). Newly generated astrocytes were double stained with green (BrdU positive) and blue (GFAP positive) (Figure 3C). Some of BrdU+ cells in green color only did not show either neural or astrocytic cell phenotype, indicating that they either were non differentiated progenitor cells or that they differentiated into other cell phenotypes not investigated here.

#### Neuronal differentiation

The percentage of double-labeled BrdU/NeuN cells was 22.4% higher in G93A-SED than vs WT-SED (80.3%±3.1% vs. 65.6%±4.3%; p = 0.007) (Figure 3D). For the WT and G93A mice, exercise training did not increase the percentage of BrdU/NeuN double-labeled cells (Figure 3E and 3F).

#### Glial differentiation

The percentage of double-labeled BrdU/GFAP cells was significantly lower by 48% in sedentary G93A vs sedentary WT mice (8.0%±6.0% vs. 15.3%±6.8%; P = 0.022), suggesting that G93A mice have a lower basal level of astrocytic differentiation as compared to WT mice. For the WT and G93A mice, exercise training did not influence the percentage of double-labeled BrdU/GFAP cells.

### mRNA Expression of Growth Factors

#### BDNF mRNA expression

**Figure 4:**
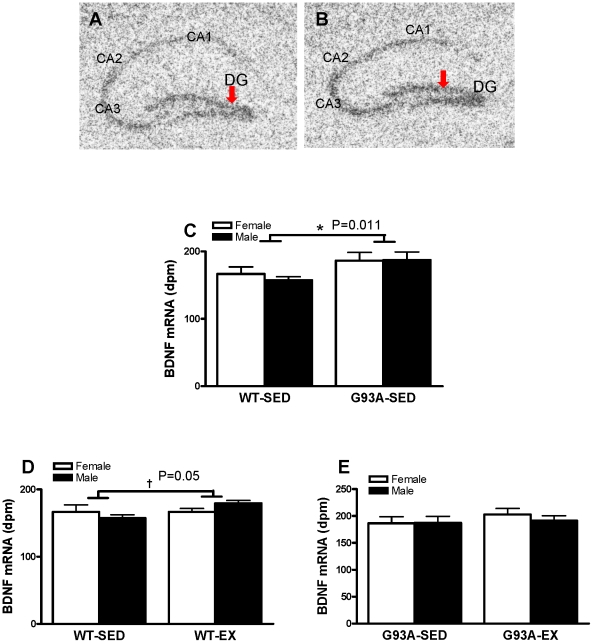
The expression of BDNF mRNA in the DG of WT and G93A mice subject to treadmill running (EX) or sedentary lifestyle (SED). A representative autoradiograph showed BDNF mRNA hybridization signal in the DG in G93A mice (B) was stronger than that in WT mice (A). (C) G93A-SED mice had higher expression of BDNF mRNA by 15.5% than WT-SED mice. * G93A-SED>WT-SED, P = 0.016, n = 10–13 per group. (D) Exercised WT mice had significantly higher BDNF mRNA expression by 7.4% vs WT-SED mice. † WT-EX>WT-SED, P = 0.05, n = 11–12 per group. (E)There was no significance between G93A-EX and G93A-SED mice, n = 10–13 per group. Data are means ± SEM. Red arrows indicated BDNF mRNA hybridization signal in the DG.

Representative autoradiographs of the hippocampal region for BDNF mRNA in WT and G93A mice are shown in Figure 4A and 4B, respectively. BDNF mRNA content in the DG was significantly higher by 15.5% in sedentary G93A vs. sedentary WT mice (186.9±8.5 dpm vs 161.8±5.6 dpm, P = 0.016) (Figure 4C). For the WT mice, exercise training increased BDNF mRNA expression by 7.4% vs. SED, with no sex difference (173.7±3.5 dpm vs 161.8±5.6 dpm; P = 0.050) (Figure 4D). For the G93A mice, there were no effects of exercise training or sex on DG BDNF mRNA expression (Figure 4E).

#### IGF1 mRNA expression

**Figure 5:**
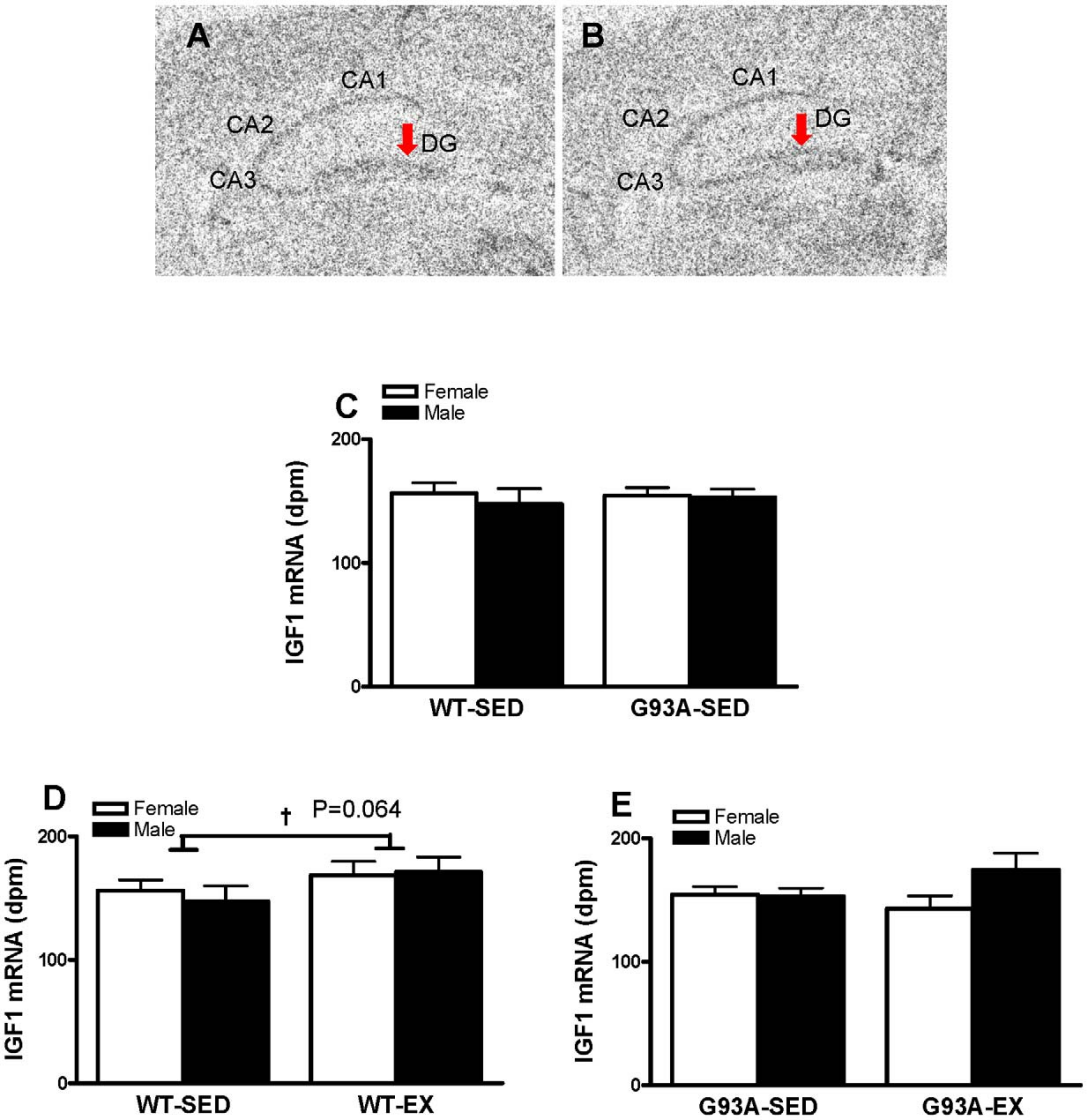
The expression of IGF1 mRNA in the DG of WT and G93A mice subject to treadmill running (EX) or sedentary lifestyle (SED). (A) IGF1 mRNA hybridization signal in the DG of WT mice. (B) IGF1 mRNA hybridization signal in the DG of G93A mice. (C) There was no significant difference between WT-SED and G93-SED mice, n = 11–13 per group. (D).Exercising WT mice had a trend to increase expression of IGF1 mRNA in the DG by 12.0% vs WT-SED mice, † P = 0.064, n = 11–12 per group. (E) There was no significant difference in DG IGF1 mRNA expression between G93A-SED and G93A-EX mice, n = 9–13 per group. Data are means ± SEM. Red arrows indicated IGF1 mRNA hybridization signal in the DG.

Representative autoradiographs of the hippocampal region for IGF1 mRNA in WT and G93A mice are shown in Figure 5A and B, respectively. Neither sex nor genotype (WT vs. G93A) influenced IGF1 mRNA expression in the DG. For the WT mice, exercise training tended to increase IGF1 mRNA by 12.0% in the DG vs. SED (170.0±8.4 dpm vs 151.8±7.6 dpm, P = 0.064) (Figure 5D). In contrast, for the G93A mice, exercise training did not lead to a significant increase in IGF1 mRNA expression in the DG (Figure 5E).

### Correlation between DG BDNF mRNA and the Survival and Neuronal Differentiation of BrdU labeled cells in G93A Mice

**Figure 6:**
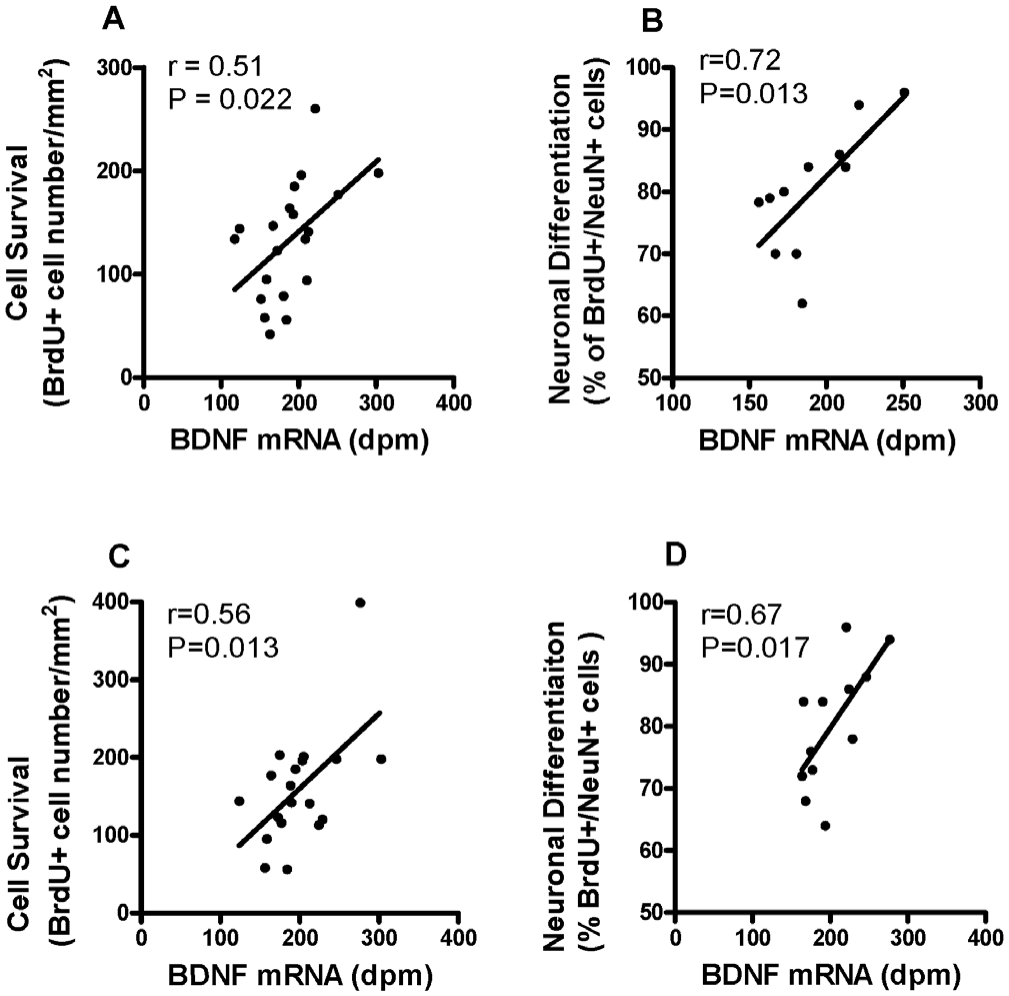
Correlation between DG BDNF mRNA and BrdU labeled surviving cells and neuronal phenotype of surviving cells in G93A mice. (A) In G93A-SED mice, DG BDNF mRNA expression was significantly and positively related to the survival of BrdU labeled cells (r = 0.51, P = 0.022, n = 20). (B) In G93A-SED mice, DG BDNF mRNA expression was significantly and positively related to the neuronal phenotype of BrdU labeled surviving cells (r = 0.72, P = 0.013, n = 11). (C) In G93A-EX mice, the expression of DG BDNF mRNA was significantly and positively related to the survival of BrdU labeled cells (r = 0.56, P = 0.013, n = 19). (D) In G93A-EX mice, DG BDNF mRNA expression was significantly and positively related to neuronal differentiation of BrdU labeled surviving cells (r = 0.67, p = 0.017, n = 11). Data are means ± SEM.

Previous studies have shown that BDNF is essential for cell proliferation, survival and differentiation in hippocampal neurogenesis [32], [66], and plays an important role in the maintenance of basal neurogenesis in the DG of adult mice [33]. DG BDNF mRNA expression was significantly and positively related to cell survival (r = 0.51, P = 0.022) (Figure 6A) and neuronal differentiation of BrdU labeled cells (r = 0.72, P = 0.013) (Figure 6B) in sedentary G93A mice. The relationship held true following exercise training with DG BDNF mRNA expression being positively related to survival (r = 0.56, P = 0.013) (Figure 6C) and neuronal differentiation of BrdU labelled cells (r = 0.67, P = 0.017) (Figure 6D).

### Oxidative Stress

Determinations of antioxidant enzymes (SOD2 and catalase) and markers of oxidative damage (3-NT and 8-OHdG) were used to evaluate the overall degree of oxidative stress. Both mRNA and protein expression levels of SOD2 and catalase in the DG for both of WT and G93A mice were examined in the present study, but there was no significant difference for any of these measurements; suggesting that G93A mice have the same baseline level of antioxidant enzymes as WT mice, and there is no effect of exercise training or sex on antioxidant enzymes in the DG region of the hippocampus in either G93A or WT mice. With regard to the extent of oxidative stress *in situ* in the DG, 3-NT as a marker of protein oxidative modification and 8-OHdG as a marker of oxidative damage to DNA were evaluated, since they are consistently reported in both ALS patients and G93A mice [60], [62], [67]

#### 3-NT Content in the DG

**Figure 7:**
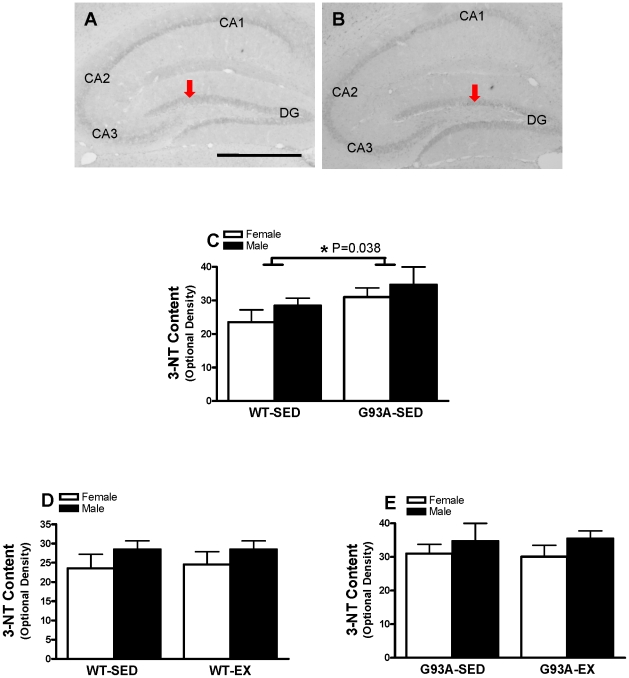
The content of nitrotyrosine (3-NT) in the DG of WT and G93A mice subject to treadmill running (EX) or sedentary lifestyle (SED). Representative black-and-white images showed 3-NT immunoreactivity in the DG of WT (A) and G93A (B) mice. (C) G93A-SED mice had 24.9% higher 3-NT content than WT-SED mice, * P = 0.038, n = 7–11 per group. (D) There was no significant difference between WT-EX and WT-SED mice, n = 9–11 per group. (E) There was no significant difference between G93A-EX and G93A-SED mice, n = 7–10 per group. Data are means ± SEM. Scale Bar = 25 µM in A, 500 µM in B,C. Red arrows indicate 3-NT immunoreactivity in the DG.

In the hippocampus, faint 3-NT immunoreactivity was detected in granule cells in the DG and pyramidal neurons in the CA1, CA2, and CA3 regions in WT and G93A mice. At high magnification, cells with 3-NT positively stained presented the granule nature. Representative images for quantitative analysis of immunostaining densitometry are shown for WT (Figure 7A) and G93A mice (Figure 7B). In SED mice, 3-NT content in the DG of G93A mice was significantly higher by 24.9% than that in WT mice (32.6±2.6 vs 26.1±2.1; P = 0.038) (Figure 7C). For the WT and G93A mice, exercise training did not influence 3-NT content in the DG (Figure 7D and 7E, respectively). These results indicated that there is excessive peroxynitrite-mediated nitration and/or NO production in the DG in G93A mice.

#### 8-OHdG Content in the DG

**Figure 8:**
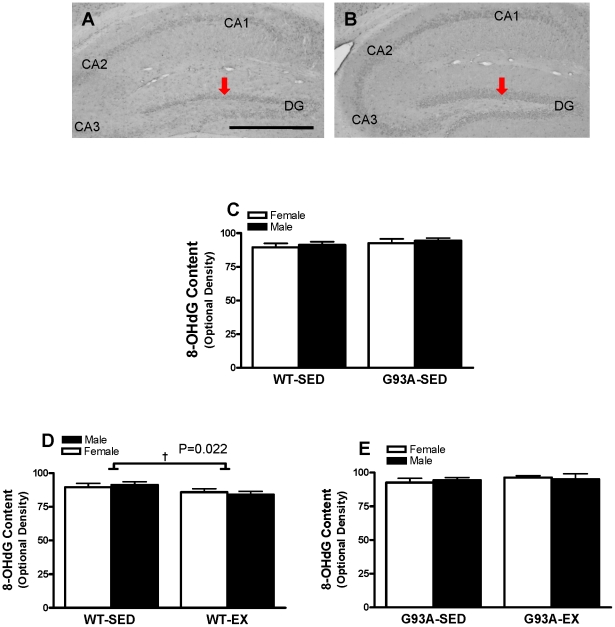
The content of 8 hydroxy-2-deoxyguanosine (8-OHdG) in the DG of WT and G93A mice subject to treadmill running (EX) or sedentary lifestyle (SED). Representative black-and-white images showed 8-OHdG immunoreactivity in the DG of WT (A) and G93A (B) mice. (C)There was no significant difference between WT-SED and G93A-SED mice, n = 11 per group except for G93A-SED males = 8. (D) WT-EX mice had 6.1% lower level of 8-OHdG than WT SED mice, † P = 0.022, n = 9–11 per group. (E) There was no significant difference between G93A-SED and G93A-EX mice, n = 8–12 per group. Data are means ± SEM. Scale Bar = 25 µM in A, 500 µM in B,C. Red arrows indicate 8-OHdG immunoreactivity in the DG.

8-OHdG immunoreactivity was detected in granule cells in the DG and pyramidal neurons in the regions of CA1, CA2, and CA3. At high magnification, 8-OHdG immunoreactivity was distributed in nuclear or peri-nuclear areas of granule cells in the DG. Representative images for quantitative analysis of optional density of staining were shown for WT (Figure 8A) and G93A (Figure 8B) mice. In SED mice, no difference in 8-OHdG immunostaining was observed between the groups (G93A vs WT) (Figure 8C). For the WT mice, exercise training lowered 8-OHdG content by 6.1% vs SED mice (84.9±1.7 vs 90.4±1.8; P = 0.022) (Figure 8D). For the G93A mice, exercise training did not influence 8-OHdG content (Figure 8E).

## Discussion

The main findings of the present study were that: (1) G93A mice had elevated basal levels of hippocampal neurogenesis (survival and neuronal differentiation of BrdU labeled cells), growth factor (BDNF) mRNA expression, and oxidative stress (3-NT) as compared to wild-type mice; (2) treadmill running promoted hippocampal neurogenesis and growth factor mRNA expression (BDNF), and lowered oxidative stress (8-OHdG) in wild type mice, with no changes in G93A mice; and (3) there was a sex difference in basal levels of hippocampal neurogenesis in G93A mice, with male G93A mice exhibiting elevated cell proliferation but reduced cell survival as compared with female G93A mice. In addition, treadmill running tended to promote cell survival in G93A female but not male mice.

### Heightened Basal Levels of Hippocampal Neurogenesis in G93A Mice

The heightened basal levels of hippocampal neurogenesis in G93A mice is in agreement with previous studies showing greater cell proliferation, migration, and neurogenesis in the spinal cord of G93A mice as compared to age-matched controls [44], [45]. Furthermore, our findings support the enhanced hippocampal-dependent spatial capability previously observed in the G93A mouse model [53]. The current results are also in line with increased expression of the IGF1 receptor, EPO, and pERK observed in the hippocampus of G93A mice [50]–[52]. IGF1, by binding to its IGF1 receptor expressed in the hippocampus, is an important modulator of brain function, ranging from neuroprotection to neural plasticity, and has been shown to induce hippocampal neurogenesis in normal rodents and is involved in hippocampal neurogenesis in a variety of animal models of brain injury, aging, and disease [68], [69]. Together, our and other studies show enhanced neurogenesis to protect against excessive levels of oxidative stress in the brain of G93A mice.

In contrast to the above findings, Liu and Martin reported reduced cell proliferation in the DG, unaltered cell proliferation in the spinal cord, and unaltered neurogenesis in both hippocampus and spinal cord in symptomatic G93A mice [43]. The discrepancy of the above findings with ours could be due to the sex of animals studied and methodological differences, including the dose of BrdU administered and the BrdU administration schedule. In their study, cell proliferation was assessed 2 h or 14 h after a single injection of BrdU at a dose of 50 µg/g body weight, and cell survival and neuronal differentiation were assessed two weeks after the last injection of BrdU at a dose of 25 µg/g, in males only. In contrast, we used daily injections of 50 µg/g for 7 consecutive days, with cell proliferation assessed 24 h after the last BrdU injection and cell survival and differentiation assessed 3 wk after the last BrdU injection (refer to Methods), in both females and males.

### Heightened Basal Levels of Growth Factors (BDNF mRNA) in G93A Mice

The higher levels of BDNF in the G93A mouse hippocampus, seen in our results, are consistent with previous studies showing higher mRNA and protein levels of BDNF in post-mortem muscle tissue of ALS patients [70], and higher BDNF mRNA in the spinal cord of G93A mice [71]. Our results are also in line with previous observations showing the activation of BDNFs downstream pathway (ERK) in the brain of G93A mice [52]. Furthermore, the higher BDNF mRNA expression in the hippocampus of G93A mice was correlated with higher cell survival and neuronal differentiation. Importantly, the up-regulation of BDNF in the hippocampus can be triggered by oxidative stress, and hence higher BDNF expression may serve as a compensatory mechanism mitigating the oxidative damage in the hippocampus of G93A mice. Indeed, oxidative stress stimulates BDNF expression, while antioxidants prevent BDNF production in neuron cell line culture [72]. Furthermore, BDNF can protect neurons from oxidative insults [73], and BDNF can acts in a positive loop with NO to inhibit neural progenitor cell proliferation and up-regulate neuronal differentiation in embryonic and adult neurogenesis [37]. Together, these findings suggest that oxidative stress and BDNF are involved in crosstalk where mutual feedback assists in regulating hippocampal neurogenesis.

The lack of an effect of the G93A genotype on IGFI mRNA content is consistent with human studies showing no differences in IGF1 immunoreactivity in the spinal cord of ALS patients vs controls [74]. In addition, DG IGF1 mRNA expression was not associated with the survival or neuronal differentiation of BrdU labelled cells (data not shown), suggesting that IGF1 was not related to elevated basal levels of cell survival or neuronal differentiation in G93A mice. This is in contrast to previous research showing that peripheral IGF1 promotes hippocampal neural progenitor cell proliferation and neuronal differentiation *in vivo* and *in vitro* [41], [68], and that it may be involved in brain injury-induced neurogenesis [68]. However, we cannot rule out the possibility that peripherally produced IGF1 may influence hippocampal neurogenesis in G93A mice.

### Heightened Basal Levels of Oxidative Stress (3-NT) in G93A Mice

An excessive level of oxidative stress in the spinal cord of G93A mice has been shown in several studies [49], [61], [75]–[79]; however, this relationship is not as strongly established in the brain of G93A mice. Chung and colleagues reported that SOD2 immunoreactivity was higher in the brain stem of G93A mice [49], and Ferrante and colleagues showed that 3-NT immuoreactivity was higher in the brain of G93A mice [26], [67], including the CA1 region of hippocampus [26]. However, no research has been conducted regarding oxidative stress in the DG. Our finding of no differences in the mRNA or protein levels for both SOD2 and catalase is consistent with previous clinical work showing no difference between ALS patients and controls in enzyme activity of SOD2 and catalase [80]. Interestingly, G93A mice have higher SOD2 protein content in the brain stem [49], spinal cord [49], [81], and skeletal muscle [82], as compared to WT mice. Furthermore, GPx activity is unaltered in the cerebellar cortex [80], but higher in the spinal cord of ALS patients as compared to controls [83]. Collectively, these studies suggest that the content of antioxidant enzymes is region-dependent in G93A mice and ALS patients.

Our finding of significantly higher 3-NT immunoreactivity in the DG of G93A mice suggests an excessive peroxynitrite-mediated nitration or NO production. This is in agreement with previous observations of high 3-NT in other regions of the brain and in the spinal cord of ALS patients and G93A mice [26], [60], [67]. Cha and colleagues evaluated 3-NT distribution in the brain of G93A mice, showing intense staining in the brain stem and cerebellum. In addition, they observed intense 3-NT immunoreactivity in the pyramidal layer, especially in the CA1 of hippocampus of G93A mice; however, they did not measure staining intensity [26]. Others have shown greater 3-NT immunoreactivity in the motor neurons of ALS patients [60] and G93A mice [67]. Our novel data suggest greater NO production in the DG of the hippocampus of G93A mice. Excessive NO generation is implicated in neuronal injury after ischemia, trauma, and neurodegenerative disorders, including ALS [84], [85].

The lack of effect of the G93A genotype on 8-OHdG suggests that the mutant SOD1 induced ROS production did not affect DNA macro-molecules in the hippocampus of G93A mice. In contrast, Aguirre and colleagues found that 8-OHdG was higher in the cortex (at age 90 and 120 days) and striatum (at age 120 days) of G93A mice as compared to age-matched littermate controls. They also found regional heterogeneity, i.e. no significant changes of 8-OHdG level in the cerebellum at any of the time points studied (at age 60, 90, and 120 days) [76]. In addition, 8-OHdG is most prominent in the ventral horn of spinal cord in ALS patients [86] and G93A mice [61]. Whether the level of 8-OHdG is altered in other brain regions in G93A mice is not clear. On the other hand, the absence of increased DNA damage in the DG of G93A mice may be due to the presence of DNA repair enzymes, such as 8-oxoguanine-DNA glycosylase (OGG1), which is a major enzyme responsible for 8-OHdG removal [87], [88]. It is possible that OGG1 is up-regulated in the DG of G93A mice, which could explain the lack of change in 8-OHdG in the DG region of the hippocampus.

### Treadmill Exercise Effect on Hippocampal Neurogenesis

Many studies and reviews have addressed the benefits of exercise on brain function [12], [89]–[92]. Exercise may improve learning and memory, postpone age-related cognitive decline, decrease the risk of neurodegenerative diseases, and alleviate depression [89], [91]–[94]. The effects of exercise are very complex and could include enhanced neurogenesis via growth factors, pulses of oxidative stress, or increased angiogenesis [58], [95], [96].

Given that oxidative stress may be a trigger for neurogenesis, we felt that the pulses of oxidative stress induced by exercise would affect hippocampal neurogenesis in the DG of both the G93A and WT mice. With respect to cell proliferation and cell survival, our results are consistent with others who have shown that treadmill exercise promoted cell proliferation and cell survival in WT mice [8], [54], [55]. However, G93A mice showed a trend for lower cell proliferation and no change in cell survival in response to exercise. In addition, treadmill exercise did not show any effect on neuronal differentiation in both WT and G93A mice. Our data are novel in showing that treadmill exercise did not affect hippocampal neurogenesis in G93A mice, yet it up-regulated hippocampal neurogenesis in WT mice, perhaps implying a negative effect of constantly elevated oxidative stress and a physiological adaptive response to “pulses” of oxidative stress in response to episodic exercise in wild-type mice.

Exercise in rodents, including wheel running or treadmill running, promotes hippocampal neurogenesis via cell proliferation and cell survival [91]. In pathological states, exercise reverses decreased hippocampal neurogenesis in murine models of normal aging, radiation, and alcohol exposure [14], [16]. However, in some animal models of neurodegenerative diseases, exercise does not induce hippocampal neurogenesis. Specifically, the amyloid precursor protein-23 model of AD showed no change in survival of newborn neurons following long-term (8 month duration) exercise [97]. In the R6/2 transgenic mouse model of HD, 4 wk of running failed to stimulate proliferation and survival of newly generated neurons [19]. Under normal conditions, exercise-induced hippocampal neurogenesis is affected by the intensity and duration of exercise [54], [98], and by the animal strain [99]. Under pathological conditions, the effect of exercise on hippocampal neurogenesis is complex. Under certain conditions, such as social isolation, exercise may increase the susceptibility to glucocorticoid-induced suppression of neurogenesis [100], [101]. Consequently, the interaction of exercise and excessive basal levels oxidative stress in the G93A mouse may have inhibited hippocampal neurogenesis.

### Treadmill Exercise Effect on Growth Factors

Growth factors may be involved in the mechanisms underlying exercise-induced hippocampal neurogenesis. We showed that treadmill exercise increased BDNF mRNA content in the DG of WT mice, which is in agreement with previous observations [9], [57], [58], [102]. In contrast, treadmill exercise did not alter BDNF mRNA expression in the DG of G93A mice, possibly due to the ‘ceiling effect’ of heightened basal levels of BDNF mRNA expression in this disease model. Although no change was observed in DG BDNF mRNA expression following exercise in G93A mice, DG BDNF mRNA was still correlated with cell survival and neuronal differentiation in G93A mice following exercise, suggesting the strong association between BDNF and cell survival and neuronal differentiation.

Treadmill exercise tended to promote higher IGF1 mRNA content in the DG of WT mice, but not in the DG of G93A mice, suggesting that central IGF1 may be involved in hippocampal neurogenesis in exercised WT mice, but not in G93A mice. Previous studies have demonstrated that peripheral IGF1 is required to mediate hippocampal neurogenesis following exercise [42], [59]. Our results suggest that central IGF1 may also be involved in exercise-induced hippocampal neurogenesis in WT mice; however, further research is warranted to confirm this observation.

### Treadmill Exercise Effect on Oxidative Stress

Exercise generates pulses of ROS due to ATP production via mitochondrial metabolism and/or the xanthine oxidase reaction. Pulses of oxidative stress following exercise lead to compensatory up-regulation of antioxidant enzymes in muscle [103], [104]. Whether exercise induces a similar response in antioxidant enzymes in the brain is not clear. In the present study, we observed no change in mRNA expression or protein level of antioxidant enzymes (SOD2 and catalase) in the hippocampus of WT or G93A mice, suggesting that treadmill exercise does not lead to compensatory antioxidant adaptation in the hippocampus of WT or G93A mice. This is in agreement with previous studies showing no alteration in antioxidant enzymes in the hippocampus following exercise [105], [106]. Cechetti and colleagues reported that daily moderate intensity exercise (20 min/d, ×2 wk of treadmill training) did not modify the level of DNA repair enzyme OGG1 in the brain [105]. Moreover, swimming training (2 h/d, 5 d/wk, ×8 wk) did not modify OGG1 enzyme activity in the rat brain [106]. In contrast, Romani found that treadmill exercise for 7.5 wk led to higher enzyme activities for SOD and GPx in the brainstem and corpus striatum of rats, but not in the hippocampus and cerebral cortex [107]. These studies suggest that the effect of exercise on antioxidant enzymes is also dependent on brain regions [107]. A recent study reported that treadmill exercise did not affect antioxidant enzyme activities of SOD1, catalase, and GPx in the brain of normal rats; whereas, it increased the activity of GPx in diabetic rats [108], suggesting the effect of exercise on antioxidant enzymes is also dependent on health vs. disease states. In summary, the response in antioxidant enzyme activity is determined by many factors, including brain region, presence of underlying disease, and mode of exercise.

The finding of lower 8-OHdG immunoreactivity in the DG of the hippocampus in WT mice is in agreement with previous observations showing that exercise training reduces oxidative damage in the brain [109]–[111], and skeletal muscle [104], [112]. However, we did not find any effect of exercise on 3-NT immunoreactivity in WT or G93A mice, suggesting that treadmill exercise training did not reduce protein nitration/damage. Paradoxically, we showed attenuated DNA oxidative damage following treadmill exercise in WT mice with no concomitant increase in antioxidant enzymes; however, we could not rule out the possibility that exercise would up-regulate the repair enzyme for oxidatively damaged DNA, i.e. OGG1, to counteract the DNA damage in the DG of the hippocampus in WT mice, or that the generation of ROS in the mitochondria or at the xanthine oxidase reaction was reduced.

### The Effect of Sex on Hippocampal Neurogenesis

Several observations led us to speculate that sex would influence hippocampal neurogenesis in G93A mice. Firstly, there are sex-based differences in hippocampal neurogenesis [56], [113]. Secondly, there are also sex-based differences in disease progression in neurodegenerative diseases including ALS [114]. Thirdly, we previously observed sex-based differences in the progression of the disease in response to exercise in G93A mice [31]. We showed that the basal level of cell proliferation was significantly higher in male vs. female G93A mice but the basal level of cell survival was higher in female vs. male G93A mice. In addition, exercised female G93A mice displayed more surviving cells than exercised male G93A mice, which is in agreement with earlier studies using exercise training in G93A mice [30], [31]. Our previous observation showed that female G93A mice have better survival and resistance to high intensity treadmill running as compared with G93A males [31]. In Veldink's study, exercise delayed the onset of disease and the total survival time in female but not in male G93A mice [30]. In contrast, we did not find sex difference in hippocampal proliferation, cell survival, or cell differentiation in WT mice. The lack of sex difference in hippocampal neurogenesis in WT mice is consistent with a recent study which failed to find evidence for sex differences in basal rates of hippocampal cell proliferation in mice [115]. Although our and Legace's studies are consistent in showing no sex difference in wild type animals, it is possible that the estrous cycle could influence the ability to detect a sex difference [20].

Taken together, G93A mice have a heightened basal level of hippocampal neurogenesis, which are associated with heightened basal levels of BDNF. Oxidative stress might indirectly affect hippocampal neurogenesis by regulating BDNF levels. Thus, in the presence of oxidative stress in the hippocampus, there is a compensatory increase in BDNF to promote hippocampal neurogenesis to protect against oxidative damage. Treadmill running has different effects on hippocampal neurogenesis, production of growth factors, and oxidative stress between G93A mice and WT mice. Treadmill exercise did promote neurogenesis, increases growth factors, and lowered oxidative stress in wild-type mice; however, it did not do so in G93A mice. In addition, we showed sex differences in basal levels of hippocampal neurogenesis and in response to exercise in G93A mice, but not in WT mice. Male G93A mice had greater cell proliferation but lower cell survival as compared with female G93A mice, and exercised male G93A mice had lower cell survival as compared to exercised female G93A mice. Whether sex differences in hippocampal neurogenesis are associated with conditions of excessive oxidative stress needs to be clarified.
